# A new approach based on targeted pooled DNA sequencing identifies novel mutations in patients with Inherited Retinal Dystrophies

**DOI:** 10.1038/s41598-018-33810-3

**Published:** 2018-10-18

**Authors:** Maitane Ezquerra-Inchausti, Ander Anasagasti, Olatz Barandika, Gonzaga Garay-Aramburu, Marta Galdós, Adolfo López de Munain, Cristina Irigoyen, Javier Ruiz-Ederra

**Affiliations:** 1 Division of Neurosciences, Biodonostia Health Research Institute, San Sebastián, Spain; 2 RETICS OFTARED, National Institute of Health Carlos III, Ministry of Economy and Competitiveness, Madrid, Spain; 3 Department of Ophthalmology, Araba University Hospital, Vitoria-Gasteiz, Spain; 4 Department of Ophthalmology, Cruces University Hospital, Bilbao, Spain; 5 Department of Neurology, Donostia University Hospital, San Sebastián, Spain; 6 CIBERNED, Center for Networked Biomedical Research on Neurodegenerative Diseases, National Institute of Health Carlos III, Ministry of Economy and Competitiveness, Madrid, Spain; 7 Department of Neuroscience, University of the Basque Country UPV-EHU, San Sebastián, Spain; 8 Department of Ophthalmology, Donostia University Hospital, San Sebastián, Spain

**Keywords:** Hereditary eye disease, Retinal diseases

## Abstract

Inherited retinal diseases (IRD) are a heterogeneous group of diseases that mainly affect the retina; more than 250 genes have been linked to the disease and more than 20 different clinical phenotypes have been described. This heterogeneity both at the clinical and genetic levels complicates the identification of causative mutations. Therefore, a detailed genetic characterization is important for genetic counselling and decisions regarding treatment. In this study, we developed a method consisting on pooled targeted next generation sequencing (NGS) that we applied to 316 eye disease related genes, followed by High Resolution Melting and copy number variation analysis. DNA from 115 unrelated test samples was pooled and samples with known mutations were used as positive controls to assess the sensitivity of our approach. Causal mutations for IRDs were found in 36 patients achieving a detection rate of 31.3%. Overall, 49 likely causative mutations were identified in characterized patients, 14 of which were first described in this study (28.6%). Our study shows that this new approach is a cost-effective tool for detection of causative mutations in patients with inherited retinopathies.

## Introduction

Inherited retinal dystrophies (IRDs) are a group of heterogeneous diseases responsible for different clinically distinctive phenotypes. The most common IRD is Retinitis Pigmentosa (RP) with a prevalence of 1 in 3500 people. RP starts with night blindness and is followed by progressive loss of peripheral vision, leading to loss of central vision and blindness in most advanced cases. Although RP is clinically distinct from other IRDs, advanced stage of RP can be difficult to distinguish from other IRDs, including cone-rod or macular dystrophies^1^. Moreover, in some cases, clinical manifestations can differ among members of the same family. IRDs can be inherited in different traits including autosomal dominant (adRP), autosomal recessive (arRP) or X-linked (XlRP). The rate of inheritance has varied across populations studied. To date, over 250 genes have been related to various IRDs and some of them are responsible for the different phenotypes observed^2^ (https://sph.uth.edu/retnet/sum-dis.htm, 3 July 2017).

Since the publication of the first draft of the human genome in 2001^3,4^, we have seen an unprecedented flourishing of sequencing technologies that provide genomic information in an accurate, fast and cost-efficient way. Methods of massive parallel sequencing such as targeted Next Generation Sequencing technologies (NGS) and Whole Exome Sequencing (WES) are the most widely used methods for the diagnosis of IRD. These methods have contributed to an exponential reduction in time and costs for the execution of the sequencing^5,6^. Nevertheless, the use of whole genome sequencing for diagnostic purposes is limited, mainly by the amount of data generated, which demands high degree of expertise in terms of big data handling and interpretation of the results, and these factors complicate its transfer to the clinicians and to the patients. Comprehensive sequencing of the coding regions of all genes (Whole Exome Sequencing or WES) is more affordable, but still has high technical requirements that are an obstacle to its use as a diagnostic method in routine clinical practice. A more practical approach for clinical diagnosis may consist of an initial genetic screening of a subset of genes associated with a phenotype using targeted NGS, followed by a second more extensive genome analysis, such as WES^6^, and the analysis of the copy number variations (CNVs)^1^, for challenging cases for which the first strategy fails to indicate any genetic explanation.

In this study, we sequenced 316 genes associated with IRDs including several syndromic retinopathies. Targeted NGS typically involves a DNA-barcode labelling of each of the individuals to be sequenced for genotyping purposes, this processing being a bottleneck process in terms of consumables, equipment and human resources. In order to simplify the sequencing process and to reduce the costs associated with individual labelling of DNA samples, we have developed a mutation detection approach based on targeted NGS in combination with high resolution melting (HRM) analysis. NGS was performed using pools of 16 DNA samples per pool, and identification of the sample/s carrying the mutation/s was performed using HRM analysis in individual samples, which allowed us to link mutations found in the pooled DNA samples to the DNA from individual patients. We sequenced samples from a total of 115 unrelated patients and 13 controls, 5 of which corresponded to samples from patients with IRD characterized by a third party laboratory. Information regarding mutations in these five controls was not revealed to us until completion of our analysis, to further test the sensitivity of our method in an objective way.

For those samples with negative results after the sequencing process, we used multiplex ligation-dependent probe amplification (MLPA) method for CNV analysis. After combining our sequencing strategy with MLPA, we were able to conclusively identify mutations in 36 patients, meaning that a genetic diagnosis rate was obtained in 31.3% of cases.

## Results

### Targeted Sequencing

A total of 316 genes (Supplementary Table S1) divided into 7222 amplicons were analysed. A total of 2864 and 3350 genetic variants were found in the 4 and 8 sample pools, respectively, while 3997 +/− 58 variants found in the 7 pools with 16 samples. Mean and median read depth obtained per sample were 196X and 193X, respectively. Less than 3.4% of targeted regions were covered less than 30X per pool, which we established as the cut off.

### Sensitivity

In order to assess the sensitivity of our method we performed two independent experiments. In the first experiment, we included a set of 3 pools all containing an increasing number of control samples prepared from DNA from 16 patients (see methodology section and Supplementary Fig. S1 for a more detailed description). Each control sample carried at least one mutation that had been previously validated by Sanger sequencing (see methodology section). As a result, previously characterized mutations from all control samples were identified in the first set of samples, regardless of the size of the pool.

Following our method, one would expect a relative level of coverage of 1/32 in heterozygous variants and 2/32 in one homozygous or in two heterozygous variants. However, we found that the number did not fit exactly to these values when analysing variants among solved patients (see variants in Table 1). Thus, in heterozygous variants the relative coverage ranged between 0.56 to 1.54/32 with 5 outliers with relative coverage of 1.75/32, 1.88/32, 1.99/32, 1.93/32 and 2/32, with values more suggestive of mutations present in two alleles rather than in one.

**Table 1:** Summary of all identified variants. Variants of uncertain significance (VUS) are in italics.

Family	Gene	Gene transcript	Allele1			Allele2			Family segregation
			cDNA Change	Protein change	Reference	cDNA Change	Protein change	Reference	
RP1	EYS	NM_001142800	c.9405T>A	p.Tyr3135Ter	^11^	c.1830del	p.His610GlnfsTer26	This study	Yes
RP8	CERKL	NM_001030311.2	c.847C>T	p.Arg283Ter	^10^	c.847C>T	p.Arg283Ter	^10^	Yes
RP15	USH2A	NM_206933	c.12093del	p.Tyr4031Ter	^8^	c.11241C>G	p.Tyr3747Ter	This study	Yes
RP17	CHM	NM_000390	c.1272_1273delinsCT	p.Gln425Ter	^41^				Yes
RP27	RPGR	NM_001034853	c.2232_2235del	p.Asp744GlufsTer70	This study				Yes
RP34	USH2A	NM_206933	c.2276G>T	p.Cys759Phe	^56^	c.5278del	p.Asp1760MetfsTer10	^8^	Yes
RP35	RP1	NM_006269	c.4804C>T	p.Gln1602Ter	^67^	c.1837dup	p.Thr613AsnfsTer6	This study	Yes
RP49	EYS	NM_001142800	c.4045C>T	p.Arg1349Ter	^12^	c.4045C>T	p.Arg1349Ter	^12^	Yes
RP57	TULP1	NM_003322	c.1495 + 1G>C		^68^	c.1495 + 1G>C		^68^	Yes
RP59	MYO7A	NM_000260	c.1200G>T	p.Lys400Asn	^69^	c.5074C>T	p.Gln1692Ter	This study	N/A
RP77	CNGA1	NM_001142564	c.301C>T	p.Arg101Ter	^70^	c.1747C>T	p.Arg583Ter	This study	Yes
RP88	MYO7A	NM_000260	c.3763del	p.Lys1255ArgfsTer8	^71^	c.6_9dup	p.Leu4AspfsTer39	This study	Yes
RP91	USH2A	NM_206933	c.11754G>A	p.Trp3918Ter	^72^	c.3669del	p.Cys1223Ter	This study	Yes
RP106	EYS	NM_001142800	c.14C>A	p.Ser5Ter	This study	c.888del	p.Lys296AsnfsTer43	This study	Yes
RP117	EYS	NM_001142800	c.4045C>T	p.Arg1349Ter	^12^	c.9405T>A	p.Tyr3135Ter	^11^	Yes
RP153	CERKL	NM_001030311.2	c.847C>T	p.Arg283Ter	^10^	c.847C>T	p.Arg283Ter	^10^	Yes
RP154	CNGA3	NM_001298	c.162_163insT	p.Arg55Ter	This study	c.162_163insT	p.Arg55Ter	This study	Yes
RP165	ABCA4	NM_000350	c.3322C>T	p.Arg1108Cys	^73^	c.3322C>T	p.Arg1108Cys	^73^	Yes
RP67	CERKL	NM_001030311.2	c.847C>T	p.Arg283Ter	^10^	c.847C>T	p.Arg283Ter	^10^	Yes
RP109	USH2A	NM_206933	c.1570G>A	p.Ala524Val	This study	c.2276G>T	p.Cys759Phe	^56^	Yes
RP141	USH2A	NM_206933	c.2276G>T	p.Cys759Phe	^56^	c.2299del	p.Glu767SerfsTer21	^74^	Yes
RP173	NR2E3	NM_014249	c.932G>A	p.Arg311Gln	^75^	c.932G>A	p.Arg311Gln	^75^	N/A
RP174	RGR	NM_001012720	c.196A>C	p.Ser66Arg	^76^	c.196A>C	p.Ser66Arg	^76^	Yes
RP175	CNGB3	NM_019098	c.1148del	p.Thr383IlefsTer13	^77^	c.852 + 1G>C		This study	Yes
RP176	CERKL	NM_001030311.2	c.847C>T	p.Arg283Ter	^10^	c.847C>T	p.Arg283Ter	^10^	Yes
RP180	USH2A	NM_206933	c.14565del	p.Asn4856MetfsTer28	This study	c.14565del	p.Asn4856MetfsTer28	This study	Yes
RP182	PDE6A	NM_000440	c.1957C>T	p.Arg653Ter	^78^	c.1705C>A	p.Gln569Lys	^79^	Yes
RP185	CNGA3	NM_001298	c.1228C>T	p.Arg410Trp	^80^	c.829C>G	p.Arg277Gly	^81^	Yes
RP196	BBS1	NM_024649	c.1220T>G	p.Met390Arg	^82^	c.1220T>G	p.Met390Arg	^82^	Yes
RP166	USH2A	NM_206933	c.14091del	p.Phe4697LeufsTer2	^7^	c.12093del	p.Tyr4031Ter	8	N/A
RP169	CERKL	NM_001030311.2	c.847C>T	p.Arg283Ter	^10^	c.356G>A	p.Gly119Asp	^83^	N/A
RP30	RP1	NM_006269	c.1625C>G	p.Ser542Ter	^84^	c.227T>C	p.Leu76Pro	This study	Yes
RP193	ABCA4	NM_000350	c.4577C>T	p.Thr1526Met	^36, 85^	c.3386G>T	p.Arg1129Leu	^86^	N/A
*RP200*	*CRB1*	*NM_201253*	*c.444_452del*	*p.Asp148_Asp150del*	^87^	*c.2843G>A*	*p.Cys948Tyr*	^88^	*Yes*
RP188	CNGA3	NM_001298	c.1228C>T	p.Arg410Trp	^80^	c.1706G>A	p.Arg569His	^81^	N/A
RP40	PRPF31	NM_015629	exons9_13deletion		This study				Yes
*RP148*	*PRPF8*	*NM_006445*	*c.6835T>G*	*p.Trp2279Gly*	*This study*				
*RP181*	*PRPF31*	*NM_015629*	*c.1165C>T*	*p.Gln389Ter*	*This study*				*No*
*RP92*	*PCDH15 CDH23*	*NM_001142763/ NM_022124*	*c.733C>T*	*p.Arg245Ter*	^89^	*c.8326G>A*	*p.Gly2776Ser*	*This Study*	*Yes*

With respect to variants expected to be in two alleles (in homozygosis in one patient or in heterozygosis in two patients), the relative coverage ranged between 1.5–2.3/32. In this case we found 4 outliers with relative levels of coverage as low as 1.25/32 (2 cases), or as high as 2.98/32 and 3.13/32. In all cases with a higher relative coverage, in relation with the number of alleles found, all the pool was Sanger sequenced individually, in order to test for the presence of another allele with that variant and we found that there were no more alleles with the mutation among the pool.

Moreover, we tested 9 SNPs with higher MAFs in order to assess if the relative level of coverage was the same in the case of having more alleles with a specific SNP within the pool. All 16 samples from the pool in which the SNP was found, were directly Sanger sequenced. Similarly to what we observed in the candidate variants, we found some variability between expected *vs*. sequenced SNPs, with a slight mismatch of the variants present according to expected values (Supplementary Table S2).

### Variant Identification

Once we established 16 as the most cost-effective sample size, we sequenced 7 pools of 16 samples/each, including a set of 19 different controls carrying a total of 21 previously detected rare (MAF < 0.003), non-causative variants (control variants). All variants selected had a MAF < 0.003 for genes mainly associated with a recessive inheritance pattern and were absent from the databases in the case of genes associated with a dominant inheritance pattern (Supplementary Table S3). As a result, all 21 control variants were also redetected. In both sets of experiments our methodology yielded 100% sensitivity.

Furthermore, we included five samples from patients with IRD provided by a third party laboratory. As information about mutations within these samples was not initially disclosed to us, we were able to use these samples as an additional way to test the sensitivity of our method. We succeeded in identifying causal mutations in all of the samples. These were: a homozygous mutation c.1645G>T (p.Glu549Ter) in the *BBS1* gene; c.1040C>A (p.Pro347Gln) mutation in the *RHO* gene; c.1703TA (p.Leu568Ter) mutation in the *CHM* gene; c.2888_2888del (p.Gly963fs) and c.3386G>T (p.Arg1129Leu) mutations in the *ABCA4* gene and a homozygous mutation, c.397C>T (p.His133Tyr) in *MYO7A* gene.

With regard to the 115 unrelated patients analysed, disease causing mutations were found in at least one allele in 61 patients. Nevertheless, since in some patients, mutations were found only in one allele in recessive genes, causal mutations were found in 36 patients, reaching a detection rate of 31.3% (Tables 1, 2 and Supplementary Fig. S2). Most of the pathogenic mutations were found in the *USH2A* gene, although in many cases only in one allele without a second mutation, and therefore in these recessive cases, we could not determine the causal mutation. Among all mutations found in characterized patients, 15 were novel, 2 missense and 13 loss-of-function (LOF) mutations. Novel missense and splicing variant mutations were potentially pathogenic, this being inferred from the score obtained from different *in-silico* tools and the fact that they co-segregated with the disease (Supplementary Table S4).

**Table 2:** Clinical features of characterized patients.

Family	Age at diagnosis	Symptoms at diagnosis	Visual Acuity in LogMAR RE	Visual Acuity in LogMAR LE	Spherical Equivalent RE	Spherical Equivalent LE	Subcapsular Cataract (Yes, No Pseudophakic)	Pale disc	Arteriolar Attenuation	Bone Spicule Retinal Pigment	Epiretinal Membrane	Macular Edema	Visual Fields (grades)	ERG (Electroretinogram)	Syndromic RP	Family member affected (including case study)
RP1	20	Photophobia	2	0.8	−2.2	−2.62	PP	Yes	Yes	Yes	No	No	No, Low Vision	Ext	No	1
RP8	17	Nyctalopia	5	5	N/A	N/A	PP	Yes	Yes	Yes	Yes	Yes	No, Low Vision	Ext	No	1
RP15	23	Nyctalopia	0.4	0.3	−0.12	−0.62	PP	Yes	Yes	Yes	Yes	No	4	N/A	No	1
RP17	26	Nyctalopia	0.7	0.1	−6.5	−5.37	No	Yes	Yes	No	Yes	Yes	4	Ext	No	2
RP27	8	Decrease VA	3	3	0.12	−0.5	PP	Yes	Yes	Yes	No	No	No, Low Vision	Ext	No	3
RP30	26	Nyctalopia	0.7	0.7	−5.5	−5.25	Yes	Yes	Yes	Yes	No	No	Altered	Ext	No	1
RP34	37	Visual Field Loss	0.3	0.8	−0.5	−0.62	PP	Yes	Yes	Yes	No	Yes	8	Ext	No	1
RP35	5	Decrease VA	0.8	1,3	0	−0.25	PP	Yes	Yes	Yes	Yes	Yes	Altered	Ext	No	1
RP40	8	Nyctalopia	0	0	0	−0.75	No	Yes	Yes	Yes	No	No	18	Ext	No	2
RP49	16	Nyctalopia	0.4	0.5	0.87	0.75	Yes	Yes	Yes	Yes	Yes	Yes	15	Ext	No	1
RP57	9	Nyctalopia	1.3	4	13	2	PP	Yes	Yes	Yes	No	No	No, Low Vision	Ext	No	1
RP59	12	Nyctalopia	0	0	1.625	−1.25	No	Yes	Yes	No	No	No	7	Ext	Usher type 1	1
RP67	50	Decrease VA	N/A	N/A	2	0.75	Yes	Yes	Yes	Yes	No	No	No, Low vision	Ext	No	2
RP77	40	Nyctalopia	0.3	0.2	0.75	0.62	PP	Yes	Yes	Yes	Yes	Yes	4	Ext	No	2
RP88	12	Visual Field Loss	1.3	1	N/A	N/A	PP	Yes	Yes	Yes	No	No	N/A, deafness	Ext	Usher type 1	2
RP91	16	Nyctalopia	0.3	0.4	−1.62	−1.87	Yes	Yes	Yes	Yes	No	No	8	Ext	Usher	1
RP106	45	Nyctalopia	4	4	−8.75	−9.5	Yes	Yes	Yes	Yes	Yes	NO	No, Low Vision	Ext	No	1
RP117	27	Decrease VA	0.5	0.4	1.12	−1.5	No	Yes	Yes	Yes	No	Yes	10	Ext	No	4
RP141	35	Nyctalopia	N/A	N/A	1	1	Yes	Yes	Yes	Yes	No	No	N/A	Ext	No	1
RP153	17	Decrease VA	3	1	−0.5	−0.25	Yes	Yes	Yes	Yes	No	No	No, Low Vision	N/A	No	2
RP154	1	Decrease VA	1	1	3	1	No	No	No	No	No	No	Central Scotoma	N/A	Achrom.	2
RP165	17	Decrease VA	3	3	N/A	N/A	Yes	Yes	Yes	Yes	No	No	No, Low Vision	Ext	No	5
RP166	N/A	Nyctalopia	0.2	0.3	−1	−1.75	Yes	Yes	Yes	Yes	No	No	7	Ext	Usher Type 2	1
RP169	31	Nyctalopia	5	4	N/A	N/A	Yes	Yes	Yes	Yes	No	No	No, Low Vision	Ext	No	2
RP173	1	Nyctalopia	1	1	−2	−0.25	No	No	No	Yes	No	Yes	No, Low Vision	Ext	No	2
RP174	38	Decrease VA	4	4	−3.37	−0.75	No	Yes	Yes	Yes	No	No	No, Low Vision	Ext	No	1
RP175	4	Decrease VA	1	1	−0.75	−0.125	No	No	No	No	No	No	No, Low Vision	*1	Achrom.	2
RP176	22	Decrease VA	0.3	0.4	−0.75	−1.5	Yes	Yes	Yes	Yes	No	No	Central scotoma	Ext	No	1
RP180	38	Nyctalopia	4	4	N/A	N/A	Yes	Yes	Yes	Yes	No	No	No, Low Vision	Ext	Usher Type 2	3
RP109	36	Nyctalopia	0.4	0.3	−0.5	0	Yes	Yes	Yes	No	Yes	Yes	7	Ext	No	1
RP182	10	Nyctalopia	0.05	0.05	−1.75	−1.25	Yes	Yes	Yes	Yes	No	No	5	Ext	No	1
RP185	1	Nystagmus	1.3	1.3	−5.37	−5.37	No	No	No	No	No	No	No, Low Vision	*1	Achrom.	1
RP196	12	Decrease VA	1	1	−1.12	−2.12	Yes	Yes	Yes	Yes	No	No	4	Ext	No	1
RP200	31	Decrease VA	0.7	3	+0.75	+1.87	No	Yes	Yes	No	No	No	No, Low Vision	Ext	No	1
RP188	49	Decrease VA	0.8	1	+7.3	+7.3	No	No	No	No	No	No	No, Low visión	C.R Ext	No	1
RP193	38	Decrease VA	1	1	+2.62	+2.61	No	No	No	No	No	No	No, Central Scotoma	N/A	No	1

Abbreviations; LE: Left eye; NA: not available; PP: Pseudophakia; RE: Right Eye; VA: Visual Acuity. *ERG not detected either in photopic nor escotopic conditions.

Regarding the distribution of mutations among our cohort of patients, most findings were found among the following five genes:

#### USH2A

Mutations within this gene were responsible for most cases of arRP in our cohort. Most of the patients were carriers of biallelic mutations. Compound heterozygous mutations are frequently reported in this gene^7,8^. Four of the mutations found in *USH2A* were novel: c.11241C>G, in patient RP15, c.3669del in patient RP91, c.1570G>A in patient RP109 and c.14565del in patient RP180. Except for patient RP180, homozygote carrier of the mutation, the rest of the patients were carriers of mutations in compound heterozygosis with the previously reported pathogenic mutations c.12093del, c.11754G>A and c.2276G>T respectively (Table 1).

#### CERKL

This was the second most commonly mutated gene in our cohort. We characterized 5 patients with the same mutation c.847C>T in this gene. In 4 of the cases it was in homozygosis and in one case it was in compound heterozygosis with c.356G>A mutation. This nonsense mutation is relatively common in Spanish cohorts^9,10^.

#### EYS

This was the third most commonly mutated gene in our cohort. Three out of four patients shared mutations, such as RP1 and RP117 with c.9405T>A^11^ and RP49 and RP117 with c.4045T>A^12^, probably indicating the sharing of a common ancestor. This finding is consistent with previous studies involving Spanish cohorts, in which *EYS* was one of the most commonly mutated genes in recessive retinitis pigmentosa^13,14^. In addition, we found three novel mutations in this gene: two frameshift mutations in compound heterozygosis c.1830del in patient RP1 and c.888del in patient RP106; and a nonsense mutation also in compound heterozygosis c.14C>A, in patient RP106.

#### RPGR

We were able to detect a novel mutation c.2232_2235del in patient RP27 in the ORF15 region of this gene. Mutations in this region are challenging to amplify due to a large segment of highly repetitive purine-rich sequences^15^. Nevertheless, the high coverage of this region we obtained using our pooled-based approach, allowed us to detect this variant (Supplementary Fig. S3).

### Variants of Uncertain Significance (VUS)

For the family RP92, two heterozygous variants were observed in *PCDH15* and *CDH23*. Despite the fact that this digenic inheritance pattern has previously been found to be causative of Usher Syndrome^16^, and that the variants segregated correctly within our family, there is some controversy with the pathogenicity of this digenism and, as far as we know, the *CDH23* and *PCDH15* digenism has been only reported in one study^16^. Despite cochlear degeneration specific to hair cells was observed in this type of mice, USH mutant mice do not display visual defects. Based on ultrastructural analyses, it has been shown that the *USH1* proteins localize at the level of microvilli-like structures, called calyceal processes, which form a collar around the base of photoreceptor outer segments. These structures have only been found in primate and other large mammals, but not in mouse photoreceptor cells^17^. This has led to propose that the absence of these structures in the mouse retina is responsible for the lack of a visual phenotype in mouse models of Usher syndrome. Regardless of this structural difference, we cannot confirm that this digenism is the causative mutation.

In the case of family RP148, a novel missense mutation c.6835T>G was found in *PRPF8* gene. The mutation was predicted to be damaging by at least 5 *in silico* predictors. Nevertheless, given the lack of a complete segregation analysis due to the unavailability of many of the samples required, we were unable to conclude that c.6835T>G is the causal adRP mutation in this family. Similarly, in family RP181, we found a novel nonsense mutation, c.1165C>T, in *PRPF31* gene. However we were not able to validate this finding in a segregation analysis due to a lack of samples available. In fact, the only family sample we were able to study was a non-affected sister who was also a mutation carrier.

### Multiplex Ligation-dependent Probe Amplification (MLPA)

**Figure 1:**
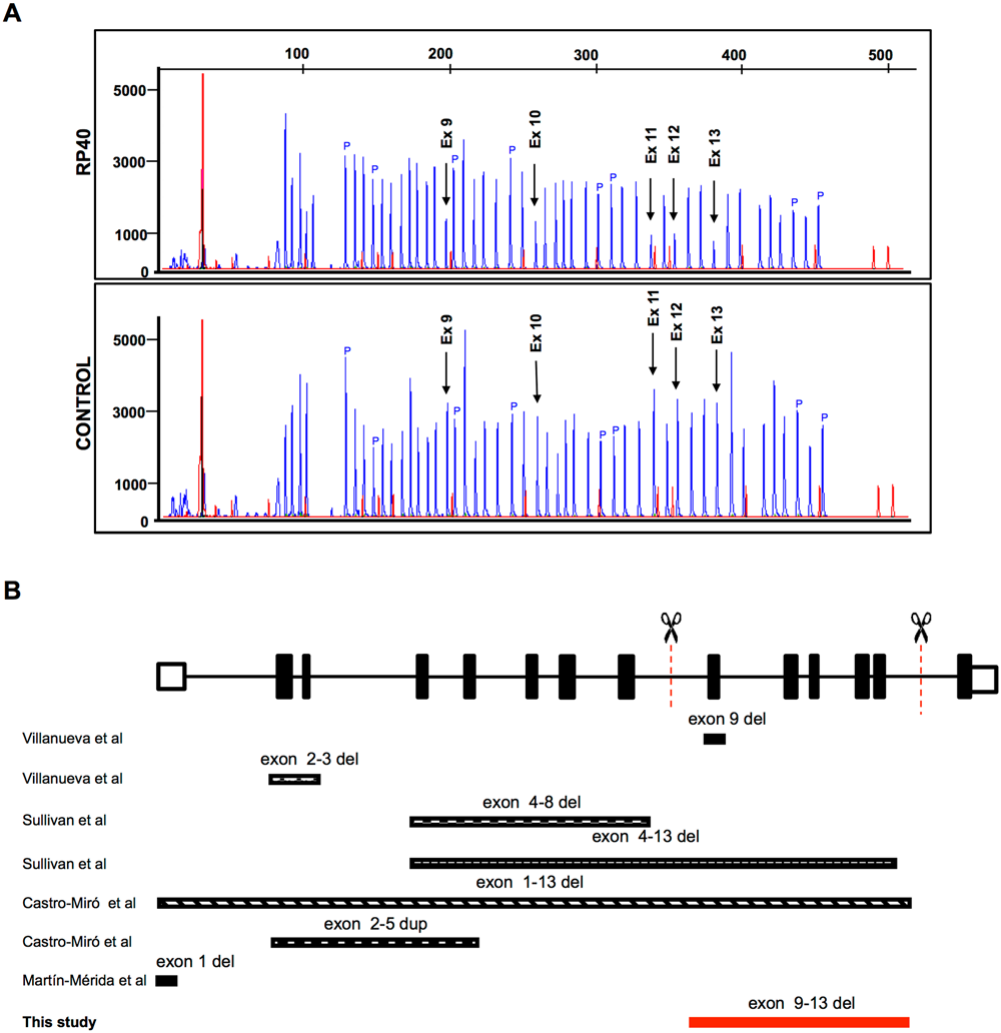
Novel deletion in *PRPF31*. (**A**) Electropherogram showing a reduced dosage of exons 9–13 (arrows) in patient RP40. (**B**) Schematic representation of *PRPF31* deletions described in the literature, and the deletion of exons 9–13 we found in this study, represented by the red bar. Abbreviations: P: control probes; Ex: Exon.

Among the 32 families analysed by this method, we detected a large deletion in the *PRPF31* gene expanding from exon 9 to 13 in family RP40, previously unreported. The deletion was also detected in an affected grandmother and the asymptomatic mother. Confirmation of the deletion region was performed sequencing the deleted DNA fragment (Fig. 1A).

## Discussion

In the present work, we have developed a cost-effective method for the diagnosis of IRDs based on pooled genomic DNA targeted NGS, in combination with HRM as a highly sensitive, versatile and affordable genotyping method. Following our methodology, we were able to find the causal mutation in 36 of our patients (31.3%) (Table 1).

Several studies have validated the feasibility of DNA sequencing pools to identify and quantify the genetic variants or single nucleotide polymorphisms (SNPs) in small genomes or small genomic regions of prokaryotes^18^; and single human genes^19,20^. Previous studies tested experimentally the accuracy in re-sequencing pools of strains of highly isogenic D. melanogaster, whose genome had been previously sequenced individually. They showed that the sequenced pool provides a correct estimate of the population allele frequency, enabling the discovery of new SNPs with a low rate of false positives^21^.

Regarding clinical applications^22^ evaluated the use of pooled DNA sequencing to accurately assess allele frequencies on transmitted and non-transmitted chromosomes in a set of families in an allelic association study^23^ combined DNA samples from 1,111 individuals and sequenced 4 genes to identify rare germline variants. The main bottleneck in the use of a pooling strategy for genetic studies is related to the challenges of detecting rare and low-frequency variants reliably, allowing an accurate estimation of MAFs^24^. Moreover, pooled DNA sequencing was applied for the analysis of 3 genes of Gitelman’s syndrome using semiconductor NGS in pooled DNA samples from 20 patients^25^. In a more recent study, 72 genes were analysed in pools consisting of samples from 12 individuals^26^. With respect to RP, pooled DNA NGS was used to search for mutations in the *SNRNP200* gene in a cohort of 96 unrelated patients from North America^27^. Pooled DNA sequencing has recently been used for population genetics studies (GWAS), in several different pathologies^28^.

Compared to previous studies that limited to the sequencing of a restricted number of genes, this represents the first study based on the pooled sequencing of more than 300 genes. To estimate the reduction in costs derived from the use of our methodology we compared the costs per patient of our pooled method with an individual sequencing approach. The main source of cost savings was related to expenses involved in the preparation of DNA libraries. Specifically, there was a 10.6-fold reduction in sequencing costs with our methodology. Once we added costs associated with the HRM analysis-based genotyping method, the overall reduction in mutation detection/patient was 6.25-fold.

The choice of 16-sample pools was based, not only on terms of sensitivity, but also on the optimal number of samples for further analysis by HRM, which we found to be around 16 in a previous study^29^. One of the main advantages over previous pooled-NGS-based strategies for mutations detection is the genotyping method we used. HRM analysis is significantly more affordable than other methods including TaqMan probes (Thermo Fisher Scientific) especially if used for a large cohort of patients and/or for a large number of genes^30^; or DNA arrays Sequenom IPLEX (CD Genomics), which requires specific equipment, making the applicability of the methodology highly dependent on the equipment available in each laboratory^30^.

In order to test the sensitivity of our method we included a set of positive controls. Five of these positive controls were samples from IRD patients previously diagnosed elsewhere, for whom we only had access to their clinical data, but not to information on the causative mutations. Given that we obtained a sensitivity of 100%, the fact that our detection rate is not as high as in previous studies, ranging from 51 to 66%^31–34^, might be explained, at least in part, by the nature of the cohort of patients included in our study, since over half of our cohort of patients (69/115) were analysed in previous studies with no results, using a repertoire of different approaches^9,29,35^.

Therefore, we believe that the great number of samples analysed in previous studies is the main factor for the relative low yield obtained. A similar observation was recently reported, where they found that the patients who were screened for the first time had a higher pathogenic variant detection rate than the overall rate, suggesting that their cohort was enriched for intractable cases giving a lower detection rate^36^.

Another possibility is that the detection rate varies depending on the ethnicity of the individuals analysed^36^. In this regard, they reported a lower rate of homozygous variants detected in individuals of European origin, comparing with other populations, in recessive transmitted diseases^36^. Similarly, we found heterozygous mutations in recessive genes in 25 patients, which therefore cannot be regarded as the causal mutation on their own. One possibility is that a fraction of our patients might be bearing large DNA re-arrangements, or mutations in deep intronic regions not covered by our approach, which would act in compound heterozygosis.

One limitation of the approach used in this work was that the relative level of coverage expected in validated variants (1/32 in heterozygous variants and 2/32 in one homozygous or in two heterozygous variants) did not fit exactly to expected values in some cases (see Results section and supplementary Table S2). This could be due to the fact that there is a pre-amplification step for library preparation. Despite great care was taken for preparing the pools using equimolar amounts of each DNA sample, we cannot discard the possibility of having some samples over or under-represented, offering higher or lower relative values, respectively. This might be reflecting an unequal sample bias, or that all DNAs of each pool were not amplified in all regions, which might be one of the potential explanations for the relative low diagnostic yield. However, we consider this possibility unlikely, considering that we were able to detect all control variants introduced in each pool.

Another limitation of pooled sequencing method is related to the lack of use of multiplex barcodes, which complicates CNV detection using NGS technology^37^.

There is increasing evidence of genomic rearrangements resulting in CNVs responsible for IRDs in several genes including *PRPF31*^38^; *EYS*^39^; *USH2A*^40^ and X-linked *RPGR* and *CHM*^41,42^. Several recent studies have emphasized the importance of CNV analysis in IRD cases. For instance, Bujakowska *et al*.^43^ found mutations in 5 out of 28 IRD cases in *SNRNP200, PRPF31, EYS* and *OPN1LW* genes. Khateb *et al*.^44^; identified rearrangements in 6 IRD patients out of 60 involving *EYS, MYO7A, NPHP4*, *RPGR* and *CHM*. This last case *CHM* was deleted in conjunction with other 6 genes. Van Cauwenbergh *et al*., 2016^1^ identified CNV in 3 patients out of 57 analysed, with mutations in *USH2A*, *HGSNAT* and *RCBTB1* genes. Interestingly, a recent paper has established a ranking of IRD genes according to genomic features and CNV occurrence. These authors recommend performing routinely a targeted CNV screening in the most prevalent 30 top-ranked IRD genes according to their genomic length^45^.

Despite some authors have described the use of read depth methods for pooled multiple sequencing^46^, we decided to select a group of 9 genes, most of which known to be prone to CNV formation^45^ using MLPA. We analysed several patients with negative results after the sequencing of the 316 IRD genes, and we included some of the genes reported as the main contributors to CNV in different studies, such as *USH2A, EYS, CHM, PRPF31* and *RPGR*^1,38,43,44,47,48^.

Using this approach, we were able to diagnose a patient with a deletion expanding from exon 9 to 13 in *PRPF31*. Rearrangements in this gene have been described to account for around 2.5% in autosomal dominant cases^38^. Although different mutated regions have been described in *PRPF31*, the deletion of exons 9 to 13 has not been described before (Fig. 1B).

The pattern of inheritance in family 40 is suggestive of an autosomal dominant pattern with incomplete penetrance. Segregation analysis was conducted in two family members, revealing the presence of an obligate carrier. Mutations in *PRPF31* have been mostly associated with cases of incomplete penetrance^49–51^.

A limitation inherent to the technique employed, which is shared by WES, is the impossibility of finding mutations in deep intronic regions, not covered by the primer design. In this regard, in an attempt to find the second mutant allele, we analysed two commonly reported deep intronic mutations: c.2991+1655A>G in *CEP290*^52^ and c.7595−2144A>G in *USH2A* genes^53,54^, in patients with heterozygous mutations in those genes. We did not however, find the mutations that were likely causative of the disease within these regions.

Despite limitations inherent to NGS sequencing regarding its performance in repetitive or CG-rich regions of the genome, we were able to detect the mutation c.2232_2235del in ORF15 of the *RPGR* gene, a region regarded as challenging, with a poor sequencing performance, both in panel based NGS and Whole exome sequencing^15^. Using our methodology we were able to detect this mutation among one of the 16 samples of the pool, which further support the validity of our method in terms of sequencing capacity, genotyping and filtering methods (Supplementary Fig. S3).

Regarding the mutations found, *USH2A* represents the most commonly mutated gene within our cohort of patients, with eleven different mutations found in this gene in seven patients characterized. Among *USH2* genes, *USH2A* is the most commonly mutated gene and it is responsible for approximately 74–90% of USH2 cases^8,55,56^. Mutations in *USH2A*, are responsible for Usher syndrome type 2 and non-syndromic RP^57^. *CERKL* and *EYS* are the next most commonly mutated genes in our cohort, which is also in accordance with previous studies^58,59^. In case of mutations in *EYS* genes, high prevalence has also been observed among Spanish population^14^, Americans with European origin^13^ and among Japanese populations^60^.

For those patients for whom we failed to identify putative disease-causing mutations, the use of alternative approaches will hopefully succeed in characterizing their disease, at the molecular level. For instance, WES aimed at the identification of mutations in genes not currently linked to IRDs; aCGH arrays for the analysis of CNVa in other genes or regions not covered by our MLPA analysis; or whole genome sequencing to extend the analysis to the 99% of non-coding DNA. Despite being highly dependent on technical support, the use of whole genome sequencing is gaining momentum in clinical practice, and it seems plausible that it will become feasible in a near future, once a robust translational genomics workflow becomes an affordable option both in economic and technical terms, to allow feedback of potentially diagnostic findings to clinicians and research participants^61^.

## Materials and Methods

### Study subjects

IRD patients were clinically diagnosed by the Ophthalmology Service at Donostia University Hospital, San Sebastian, Spain. Most patients studied had been given a diagnosis of retinitis pigmentosa, though a few patients with an undetermined inherited retinal dystrophy (IRD) were also included, based on pedigrees and clinical criteria. The inclusion criteria used were night blindness, peripheral visual field loss, pigmentary deposits resembling bone spicules, retinal vessels attenuation, optic disc pallor and reduced rod and cone response amplitudes and a delay in their timing in the electroretinogram (Hartong, 2006). A total of 115 probands were selected. In addition, samples from 13 patients were included as characterized control patients. This control group was composed of 8/13 samples selected from our cohort of IRD patients with mutations identified in previous studies^9,29,35^ and a further 5 control samples from IRD patients characterized by a third party laboratory, (those for which we were blinded to information regarding mutations until we had completed our analysis). Family pedigrees were generated from information obtained from probands. All procedures performed in studies involving human participants received approval from the ethical standards of the Clinical Research Ethics Committee of the Basque Country, Spain (CEIC-E) and were in accordance with the 2013 Helsinki declaration or comparable ethical standards. Informed consent was obtained from all individual participants included in the study.

### Human sample collection

High molecular weight DNA was extracted from blood samples from RP patients and their available family members. Total DNA from samples was extracted and isolated with the AutoGenFlex Star instrument (AutoGen, Holliston, MA, USA) using the FlexiGene DNA Kit (Qiagen, Hilden, Germany) following the manufacturer´s instructions. DNA concentrations were measured on the Qubit fluorometer using Quant-iT PicoGreen reagent (Invitrogen, Thermo Fisher Scientific, Waltham, MA, USA). Equimolar amounts of DNA samples were pooled (100 ng/ul per sample). For a detailed description of the procedure see^29^.

### Pooled sequencing

In order to assess the sensitivity and cost-effectiveness of our method we performed a first experiment to compare the yield obtained after sequencing pools with increasing number of DNA samples and we estimated the differences in costs involved in individual *vs*. pooled sequencing. All pools were made up from samples from carriers of low-frequency variants, which corresponded to either causal, variants of uncertain significance (VUS) or non-pathogenic variants identified in previous studies^9,29,35^. A total of 13 control samples were used in 3 sets of pools, with 4, 8 and 16 control samples in each. Of these control samples, 9 carried pathogenic variants (one provided by a third party laboratory), while 7 carried low frequency variants with a minor allele frequency (MAF) <0.003, and therefore we used these 7 samples both as controls and as test samples. Samples were prepared as follows: An initial pool of 4 samples was generated. This pool was used to generate the 3 pools, adding 0, 4 or 8 more samples to generate the pools with 4, 8 and 16 samples, respectively (Supplementary Fig. S1A and Supplementary Table S3A).

In order to further test the sensitivity of our method and to detect possible differences in the sequencing yield, inherent to each sequencing run, we conducted a complementary experiment. For this, we used a different set of controls, all from carriers of low-frequency, non-disease causing variants or individuals with recessive phenotypes with disease causing mutations present in only one allele. In this case, out of 115 patients analysed, a total of 108 test samples were interrogated: 16/108 corresponded to carriers of a total of 21 previously detected non disease causing variants with low MAF (<0.003) and were, therefore, used as both control and test samples (Supplementary Table S3B). 53/108 samples corresponded to patients that had been interrogated previously with negative results, and 39/108 corresponded to new samples interrogated in this study for the first time. As additional controls we used four samples from carriers of disease causing mutations provided by a third party laboratory (for which we were blinded to mutation-related information until after our analysis). For this experiment, patients were divided into 7 pools with 16 samples each. Control samples were distributed among each pool such as that each pool contained at least 2 control samples, and 4/7 pools had also control from a third party laboratory (Supplementary Fig. S1B).

### Amplicon Library preparation

Ion AmpliSeq Library Preparation Kit v2.0 (Thermo Fisher Scientific) was used to construct an amplicon library from genomic target regions with a maximum read length of approximately 200 base pairs (average length, 142 bp) for shotgun sequencing on an Ion Proton system (Thermo Fisher Scientific). Briefly, target genomic regions were amplified by simple PCR using Ion Ampliseq primer pools and 10 ng of each DNA samples.

### Sequencing Analysis

#### Ion Proton Sequencing

NGS was carried out on the Ion Proton system (Thermo Fisher Scientific). Briefly, enriched ion sphere particles (ISPs) were annealed with the sequencing primer and mixed with the sequencing polymerase from the Ion PGM_200 Sequencing Kit (Thermo Fisher Scientific). Then, the polymerase-bound and primer-activated ISPs were loaded into the previously checked and washed Ion PI Chips (Life Technologies) and having planned the run on the Ion Proton System software, chips were subjected to 500 cycles of sequencing with the standard nucleotide flow order. Signal processing and base calling of data generated from the Ion Proton runs were performed with the Ion Torrent platform-specific analysis software (Torrent Suite version 4.0).

#### Variant calling

Using the Ion Reporter software we performed the variant calling. First of all GRCh37/hg19 was used as reference genome and alignment was performed against a bed file containing all regions corresponding to 316 genes sequenced. A key aspect in our mutation detection pipeline was to take into consideration the *dilution* effect of each variant due to our pooled sequencing approach. Therefore we used the pipeline provided by the ion reporter program for the detection of somatic mutations with minor modifications. We used a somatic mutation detection approach, since this is the most suited for the detection of variants represented in very low frequency (1 in 32 alleles, in the lowest case). The only modification to the default parameters provided by the ion reporter program (5.0 version) consisted on the switch of 10 parameters within the Variant Filtering section in Parameters tab. All parameters are described in detail in Supplementary Table S5. Finally, a Variant Caller File (VCF) was generated.

### Genotyping by high resolution melting (HRM) analysis

**Figure 2:**
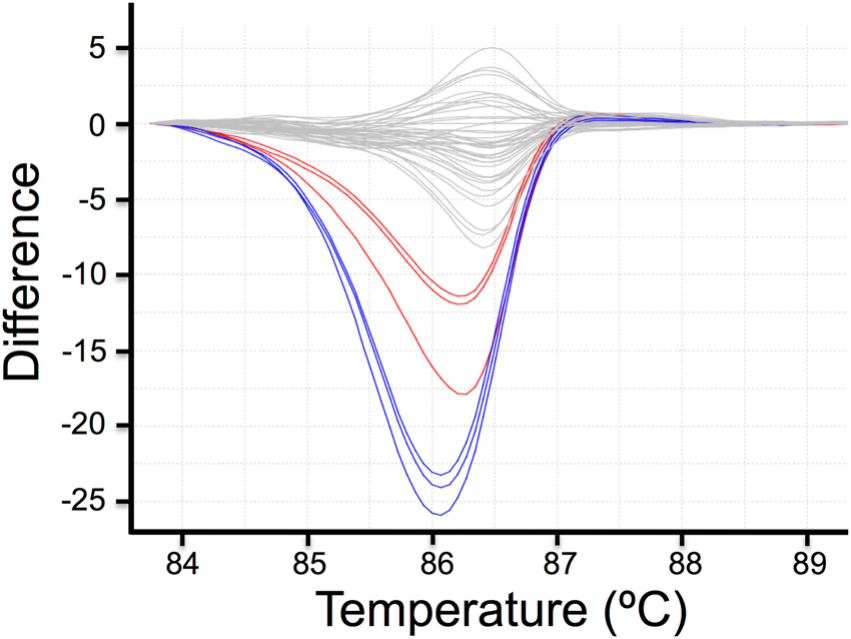
HRM analysis of *TULP1* gene. Difference plot shows c.1495+1G>C mutation in *TULP1* gene, with 2 out of 16 samples that clearly differ from the non-carrier samples (grey lines). Sanger sequencing confirmed the presence of the mutation c.1495+1G>C in two patients, one in heterozygosis (blue lines) and the other one in homozygosis (red lines). Note that samples are in triplicates.

Likely disease causing variants from each pool of 16 samples were selected from the VCF. Specific primers were designed to perform a HRM analysis generating amplicons ranging between 250 to 330 bp in length, in order to cover the mutation position. HRM analysis was used to identify which sample/s among 16 in the pool carried the mutation. We followed the methodology described in^29^, with minor modification. Briefly, PCR amplification and HRM were performed in a single run on a 7900HT Fast Real-Time PCR System in 384-well plates (Applied Biosystems), each plate contained individual samples (in triplicates) from the 16 probands of the pool in which the variant was detected. We analysed up to 7 different variants in parallel in a single run. After HRM run, the analysis of post amplification fluorescent melting curves was performed using the HRM V2.0.1 software (ThermoFisher Scientific). Melting curves were normalized and difference plots were generated to compare the samples. Only samples showing a different melting curve (Fig. 2) were Sanger sequenced.

### Sanger sequencing

Sanger sequencing was used to confirm those mutations detected by NGS and for co-segregation analysis using a 16-capillary ABI 3130xl platform (Applied Biosystems, Foster City, CA, USA) according to manufacturer´s protocol. Sequences were analysed and compared with wild-type samples and a reference sequences using BioEdit software (Ibis Biosciences, Carlsbad, CA, USA) and Ensembl and NCBI databases.

### Relevant variant priorization and pathogenicity score

In order to determine genomic variants of relevance, we selected the potential disease causing variants according to the following pre-established criteria:

1. Variants previously reported as pathogenic.
2. Variants with a MAF <0.001 for dominant genes or MAF <0.003 for recessive genes obtained from genome aggregation database (gnomAD).
3. Novel Splicing variants and loss-of-function variants such as nonsense mutations, frameshift deletions or insertions.
4. Previously reported missense variants with pathogenicity scores assessed by *in silico* predictive software.
5. Novel missense variants predicted to be damaging by *in-silico* predictive software (as mentioned below).

Presence for all candidate variants was checked using the Spanish Variant Server Database (CSVS), (http://csvs.babelomics.org/)^62^. For dominant variants, only those absent from this database were considered further. With regard to recessive variants, only those variants with a MAF lower than 0.003 and only present in heterozygosis were considered further.

### Multiplex Ligation-dependent Probe Amplification assay (MLPA)

MLPA was used to search for genomic copy number variations in 32 patients without causative mutations found after sequencing of 316 IRD genes. We selected 9 genes with high prevalence of reported rearrangements^38–40^.

Patients with a dominant inheritance pattern were analysed using MLPA Retinitis Probemix (P235). This probemix contains *PRPF31*, *RHO*, *RP1* and *IMPDH1* genes.

Patients with heterozygotic mutations in *USH2A* genes or *EYS* were also analysed for CNVs, in search of the second mutated allele within these genes (Salsa Mixes P361/2 and P328, respectively).

In addition, patients with an X-linked inheritance pattern, clinically diagnosed with choroideremia or families with only males affected, were analysed for *RP2*, *RPGR* and *CHM* genes (Salsa probemix P366).

MLPA reactions were run according to the manufacturer’s general recommendations (MRC-Holland, Amsterdam, Holland) as previously described^63^. The MLPA reaction products were separated by capillary electrophoresis on Abi Prism 3130XL Analyzer (Applied Biosystems) and the results obtained were analysed by GeneMapper software (Thermo Fisher Scientific).

### Pathogenicity predictive software

SIFT (http://www.sift.bii.a-star.edu.sg).

Polyphen2 (http://www.genetics.bwh.harvard.edu/pph2/).

PROVEAN (http://provean.jcvi.org/seq_submit.php)^64^.

GVGD (agvgd.iarc.fr/agvgd_input_php)^65^.

MutationTaster (www.mutationtaster.org)^66^.

### Web sources

Ensembl, http://www.ensembl.org/.

NCBI, http://www.ncbi.nlm.nih.gov/.

Polyphen-2, http://www.genetics.bwh.harvard.edu/pph2/.

RetNet, http://www.sph.uth.tmc.edu/Retnet/.

SIFT, http://www.sift.bii.a-star.edu.sg/.

SNPnexus, http://www.snp-nexus.org/.

The Human Genome Variation Society (HGVS), http://www.hgvs.org/.

1000 Genomes, http://www.1000genomes.org/_ENREF_48.

NHLBI Exome Sequencing Project (ESP), http://evs.gs.washington.edu/EVS/.

Babelomics, http://csvs.babelomics.org.

ExacBrowse, http://exac.broadinstitute.org/.

GnomAD browser, http://gnomad.broadinstitute.org/.

## Electronic supplementary material

SUPPLEMENTARY INFORMATION
