# Author Correction: A new approach based on targeted pooled DNA sequencing identifies novel mutations in patients with Inherited Retinal Dystrophies

**DOI:** 10.1038/s41598-019-44425-7

**Published:** 2019-05-28

**Authors:** Maitane Ezquerra-Inchausti, Ander Anasagasti, Olatz Barandika, Gonzaga Garay-Aramburu, Marta Galdós, Adolfo López de Munain, Cristina Irigoyen, Javier Ruiz-Ederra

**Affiliations:** 1 Division of Neurosciences, Biodonostia Health Research Institute, San Sebastián, Spain; 2 RETICS OFTARED, National Institute of Health Carlos III, Ministry of Economy and Competitiveness, Madrid, Spain; 3 Department of Ophthalmology, Araba University Hospital, Vitoria-Gasteiz, Spain; 4 Department of Ophthalmology, Cruces University Hospital, Bilbao, Spain; 5 Department of Neurology, Donostia University Hospital, San Sebastián, Spain; 6 CIBERNED, Center for Networked Biomedical Research on Neurodegenerative Diseases, National Institute of Health Carlos III, Ministry of Economy and Competitiveness, Madrid, Spain; 7 Department of Neuroscience, University of the Basque Country UPV-EHU, San Sebastián, Spain; 8 Department of Ophthalmology, Donostia University Hospital, San Sebastián, Spain

Correction to: *Scientific Reports* 10.1038/s41598-018-33810-3, published online 18 October 2018

The original version of this Article contained errors.

The Article contained a typographical error in the spelling of the author Gonzaga Garay-Aramburu, which was incorrectly given as Gonzaga Garai-Aramburu.

Furthermore, the Acknowledgements section in this Article was incomplete.

“This work was supported by grants from the National Institute of Health Carlos III (Institute of Health Carlos III/ISCIII) (CP10/00572, PI13/02621 and RD16/0008/0027 to JRE, PI17/01413 to CI, and a Research Intensification Contract to ALdM); the Basque Government’s Industry Department (SAIOTEK: SAIO11-PE11BN002; and SAIO12-PC12BN001 to JRE), a grant from the Mutua Madrileña Foundation and support from the Retinitis Pigmentosa Patients of Gipuzkoa Foundation (BEGISARE). JR-E is a Miguel Servet II Fellow, National Institute of Health Carlos III (ISCIII). MEI was supported by grants from the Basque Government’s Department of Education (DEDUC14/309). OB is supported by funding from the Retinitis Pigmentosa Patients of Gipuzkoa Foundation (BEGISARE) and a grant from the Mutua Madrileña Foundation. AA was supported by grants from the Fundación Jesús de Gangoiti Barrera and from the Basque Government’s Departments of Industry and Education (SAIOTEK-11BN002/PC12BN001/DEPLC13/002). CI is partially supported by a Research Intensification Contract (INTBIO15/001). The authors are grateful to Xabier Elcoroaristizabal and Marta Fernández-Mercado for their helpful advice on developing the base-calling setup. Maribel Gómez; Naiara Telletxea and Nahikari Pastoriza at the Basque Biobank for isolating DNA samples; and Dr. Carmen Ayuso for kindly providing control samples. We also give special thanks to all patients with IRD and their families involved in the study”.

now reads:

“This work was supported by grants from the National Institute of Health Carlos III and the European Regional Development Fund (Institute of Health Carlos III/ISCIII) (CP10/00572, PI13/02621 and RD16/0008/0027 to JRE, PI17/01413 to CI, and a Research Intensification Contract to ALdM); the Basque Government’s Industry Department (SAIOTEK: SAIO11-PE11BN002; and SAIO12-PC12BN001 to JRE), a grant from the Mutua Madrileña Foundation and support from the Retinitis Pigmentosa Patients of Gipuzkoa Foundation (BEGISARE). JR-E is a Miguel Servet II Fellow, National Institute of Health Carlos III (ISCIII). MEI was supported by grants from the Basque Government’s Department of Education (DEDUC14/309). OB is supported by funding from the Retinitis Pigmentosa Patients of Gipuzkoa Foundation (BEGISARE) and a grant from the Mutua Madrileña Foundation. AA was supported by grants from the Fundación Jesús de Gangoiti Barrera and from the Basque Government’s Departments of Industry and Education (SAIOTEK-11BN002/PC12BN001/DEPLC13/002). CI is partially supported by a Research Intensification Contract (INTBIO15/001). The authors are grateful to Xabier Elcoroaristizabal and Marta Fernández-Mercado for their helpful advice on developing the base-calling setup. Maribel Gómez; Naiara Telletxea and Nahikari Pastoriza at the Basque Biobank for isolating DNA samples; and Dr. Carmen Ayuso for kindly providing control samples. We also give special thanks to all patients with IRD and their families involved in the study”.

These errors have now been corrected in the PDF and HTML versions of the Article, and in the accompanying Supplementary Information file.

