# Supplementary material for: A new approach based on targeted pooled DNA sequencing identifies novel mutations in patients with Inherited Retinal Dystrophies
Source: Sci Rep. 2018 Oct 18;8:15457. doi: 10.1038/s41598-018-33810-3 (PMC6194132; doi:10.1038/s41598-018-33810-3)
Supplement: Supplementary file 1 — SUPPLEMENTARY INFORMATION [file 41598_2018_33810_MOESM1_ESM.pdf]

Supplementary Information

A new approach based on targeted pooled DNA sequencing identifies novel mutations in patients with Inherited Retinal Dystrophies.

Maitane Ezquerra-Inchausti [1,2], Ander Anasagasti[1], Olatz Barandika[1], Gonzaga Garay-Aramburu[3], Marta Galdós[4], Adolfo López de Munain[1,5,6,7], Cristina Irigoyen[1,8*], Javier Ruiz-Ederra[1,2*].

[1] Division of Neurosciences, Biodonostia Health Research Institute, San Sebastián, Spain; [2] RETICS OFTARED, National Institute of Health Carlos III, Ministry of Economy and Competitiveness, Spain [3]Department of Ophthalmology, Araba University Hospital, Vitoria Gasteiz, Spain; [4]Department of Ophthalmology, Cruces University Hospital, Bilbao, Spain [5]Department of Neurology, Donostia University Hospital, San Sebastián, Spain; [6]CIBERNED, Center for Networked Biomedical Research on Neurodegenerative Diseases, National Institute of Health Carlos III, Ministry of Economy and Competitiveness, Spain; [7]Department of Neurosciences, University of the Basque Country UPV-EHU, Spain; [8]Department of Ophthalmology, Donostia University Hospital, San Sebastián, Spain.

Correspondence: Javier Ruiz-Ederra, Ph.D., Department of Neurosciences, Biodonostia Health Research Institute, Paseo Dr. Begiristain s/n, E-20014-San Sebastián, SPAIN, Phone: (+34) 943-006128; Fax: (+34) 943006250.
Email:javiruizederra@yahoo.es/javier.ruizederra@osakidetza.eus.
Cristina Irigoyen, MD, Ph.D.; Department of Ophthalmology, Donostia University Hospital, San Sebastián, Spain, Phone: (+34) 943-006128; Fax: (+34) 943006250
Email: cristina.irigoyenlaborra@osakidetza.eus.

Supplementary Figure 1

Schematic representation of sample preparation in two sets of experiments. A. DNA was pooled in groups of 4, 8 or 16. **B.** Seven pools with 16 samples each were prepared.
In both cases lines represent DNA from 1 patient. **Green** and **blue** lines correspond to samples from patients previously characterized by our group (green) or by a third laboratory (blue). **Red** lines correspond to samples from unsolved patients, carriers of at least 1 low frequency variant (MAF<0,003), and therefore were used both as control and test samples. **Black** lines correspond to either new samples or samples with no rare variants found in previous studies. Information from variants used as positive controls is described in Supplementary Table S1.

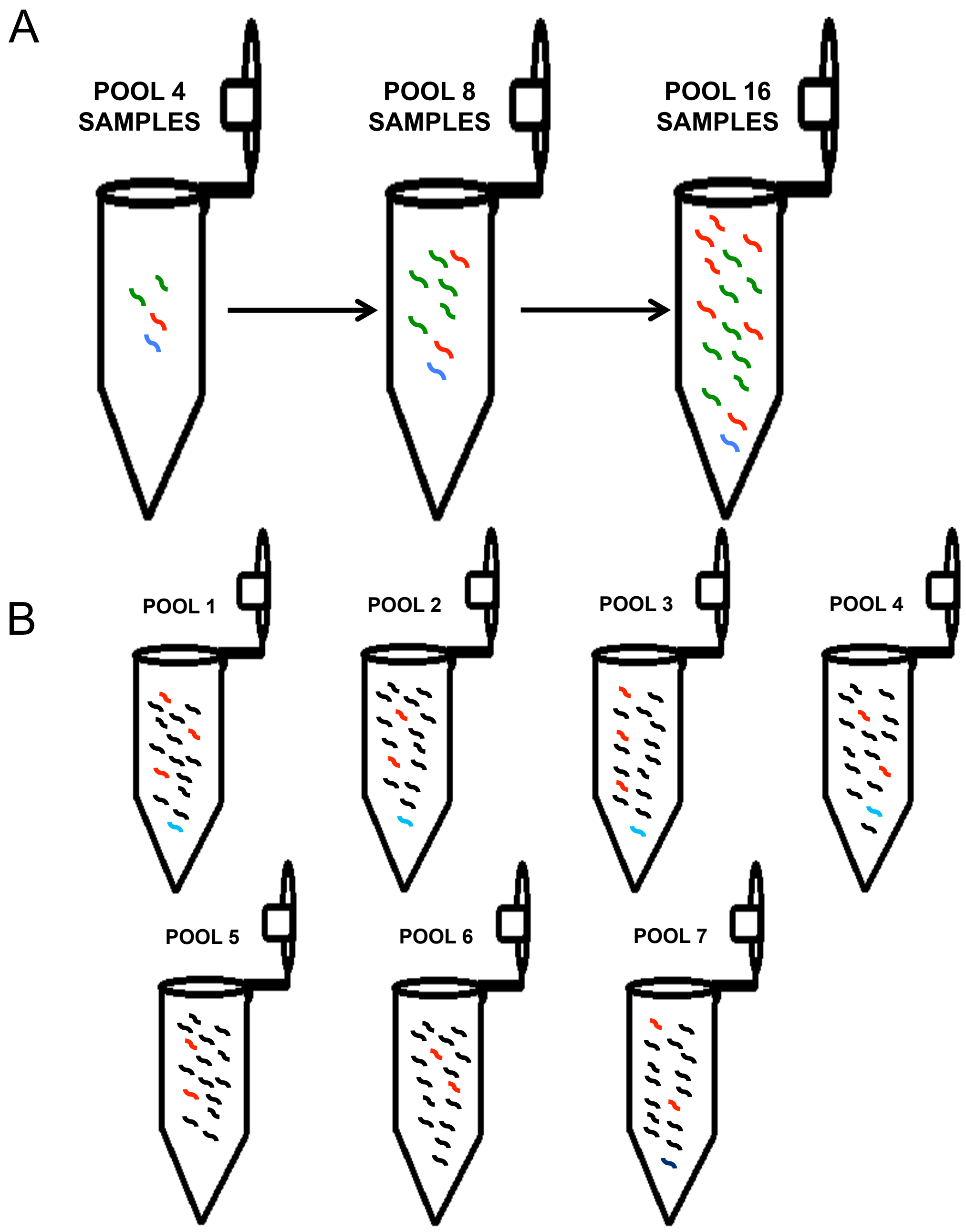

Supplementary Figure 2

Family trees from all available probands with causal mutations and with variants of uncertain significance (VUS). Genotypes are annotated as M/M or M1/M2 (homozygotes or compound heterozygotes); M/+, M1/+ or +M2 (heterozygotes); or +/+ (wild type). Arrow indicates probands.

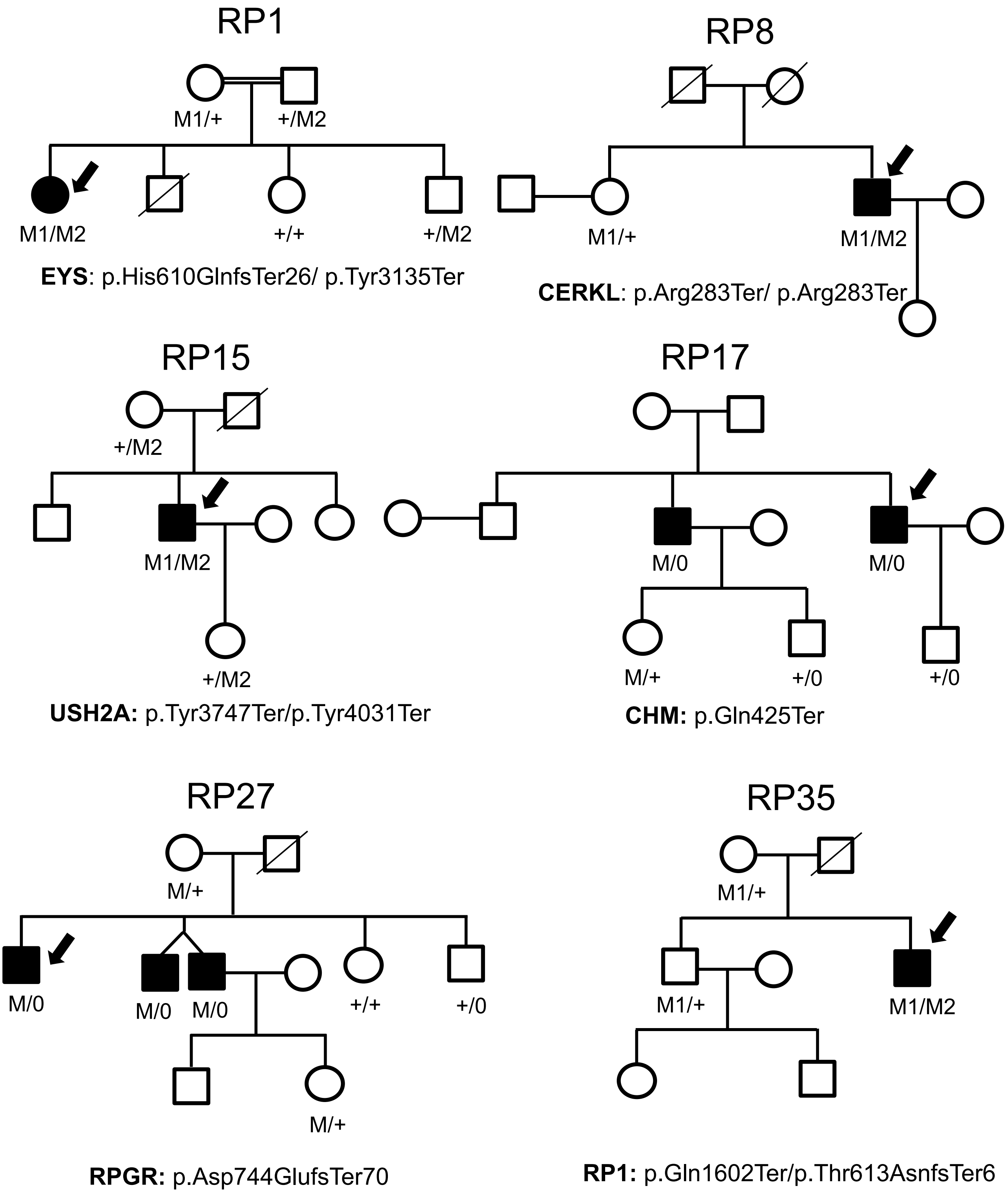

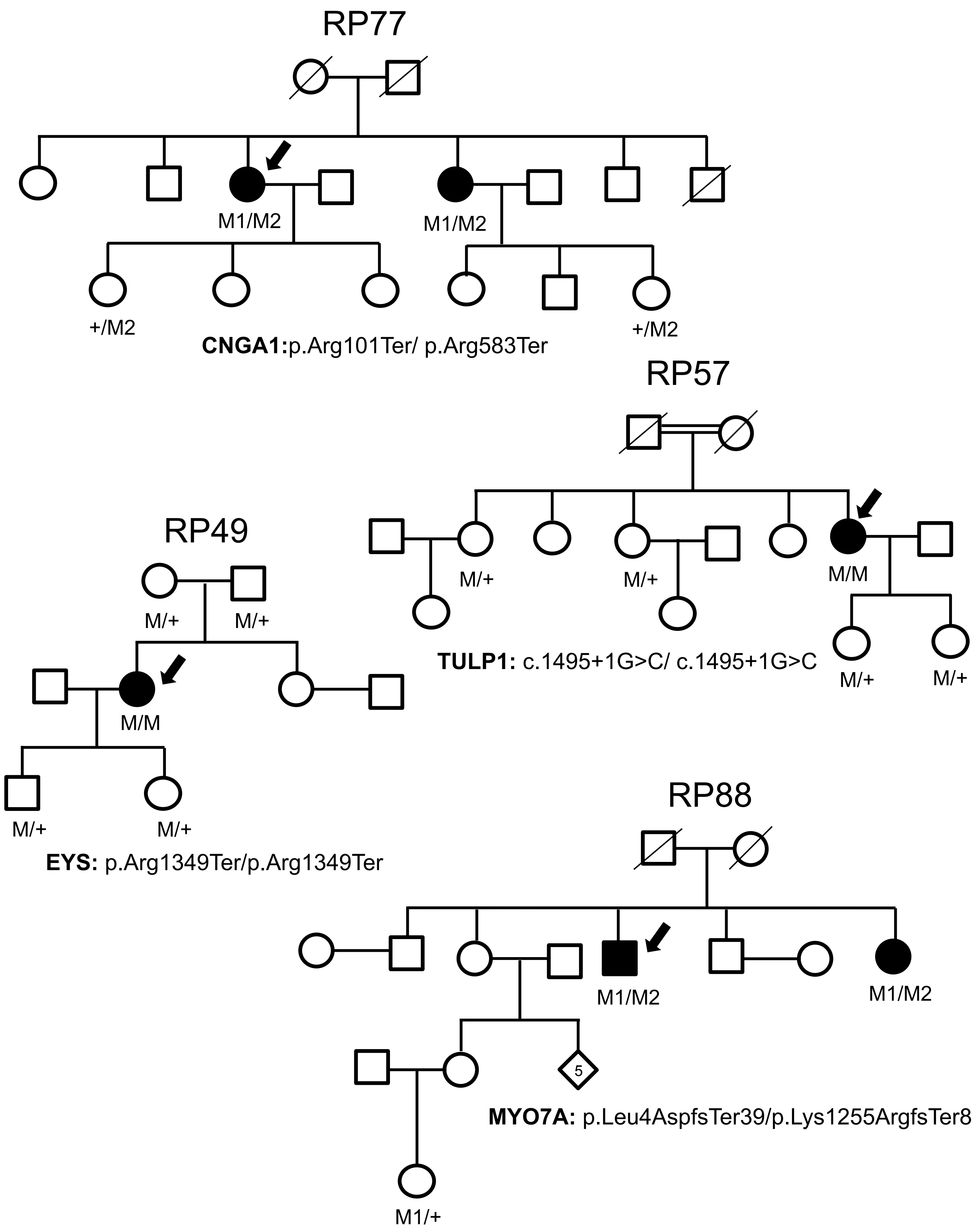
RP77
M1/M2
M1/M2
+/M2
+/M2
CNGA1:p.Arg101Ter/ p.Arg583Ter
RP57
M/+
M/+
M/M
M/+
M/+
TULP1: c.1495+1G>C/ c.1495+1G>C
RP49
M/+
M/+
M/M
M/+
M/+
EYS: p.Arg1349Ter/p.Arg1349Ter
RP88
M1/M2
M1/M2
5
MYO7A: p.Leu4AspfsTer39/p.Lys1255ArgfsTer8
M1/+

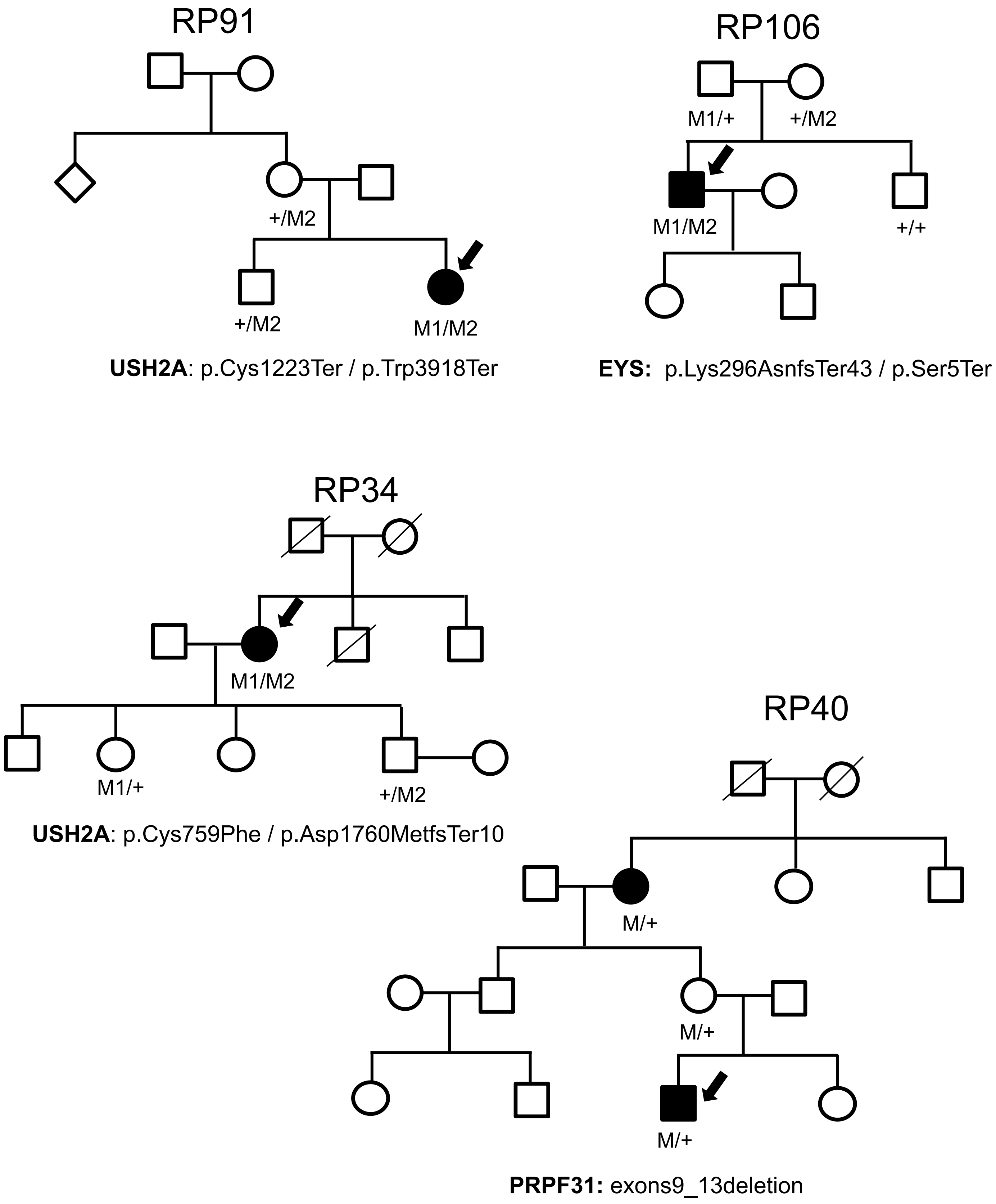
RP91
+/M2
+/M2
M1/M2
USH2A: p.Cys1223Ter / p.Trp3918Ter
RP106
M1/+
+/M2
M1/M2
+/+
EYS: p.Lys296AsnfsTer43 / p.Ser5Ter
RP34
M1/M2
M1/+
+/M2
USH2A: p.Cys759Phe / p.Asp1760MetfsTer10
RP40
M/+
M/+
M/+
PRPF31: exons9_13deletion

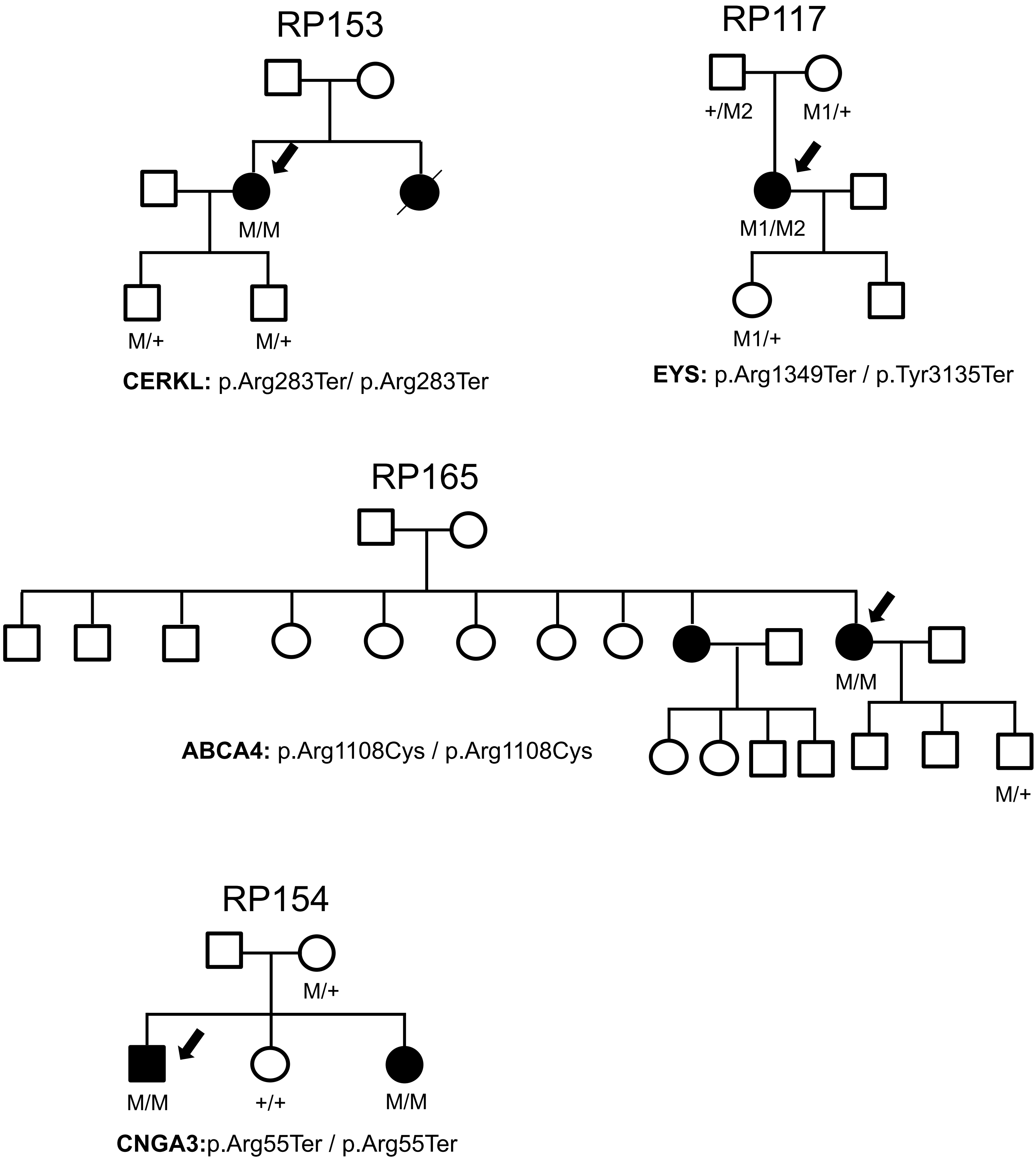
RP153
M/M
M/+
M/+
CERKL: p.Arg283Ter/ p.Arg283Ter
RP117
+/M2
M1/+
M1/M2
M1/+
EYS: p.Arg1349Ter / p.Tyr3135Ter
RP165
M/M
M/+
ABCA4: p.Arg1108Cys / p.Arg1108Cys
RP154
M/+
M/M
+/+
M/M
CNGA3:p.Arg55Ter / p.Arg55Ter

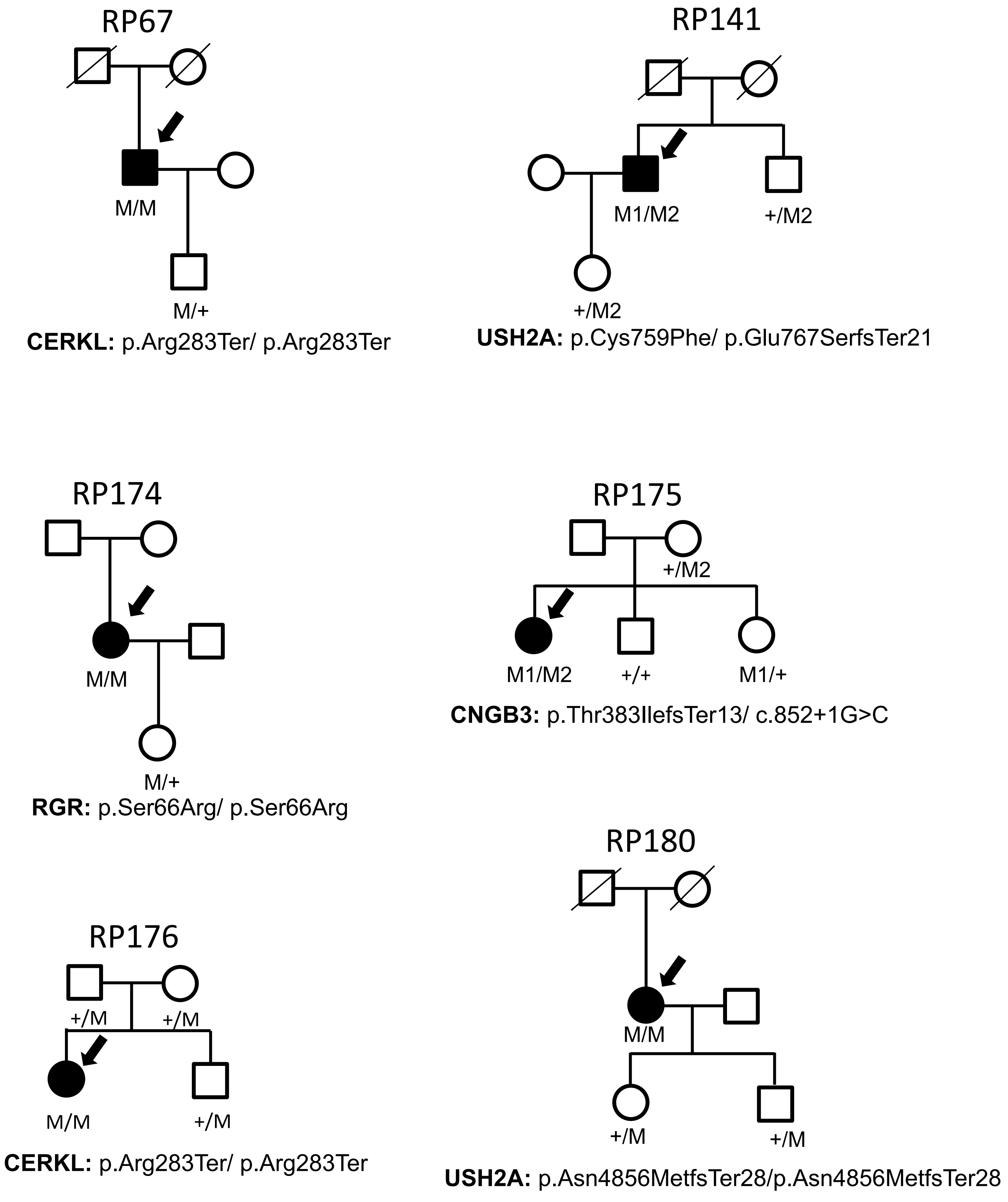
RP67
M/M
M/+
CERKL: p.Arg283Ter/ p.Arg283Ter
RP141
M1/M2
+/M2
+/M2
USH2A: p.Cys759Phe/ p.Glu767SerfsTer21
RP174
M/M
M/+
RGR: p.Ser66Arg/ p.Ser66Arg
RP175
+/M2
M1/M2
+/+
M1/+
CNGB3: p.Thr383IlefsTer13/ c.852+1G>C
RP176
+/M
+/M
M/M
+/M
CERKL: p.Arg283Ter/ p.Arg283Ter
RP180
M/M
+/M
+/M
USH2A: p.Asn4856MetfsTer28/p.Asn4856MetfsTer28

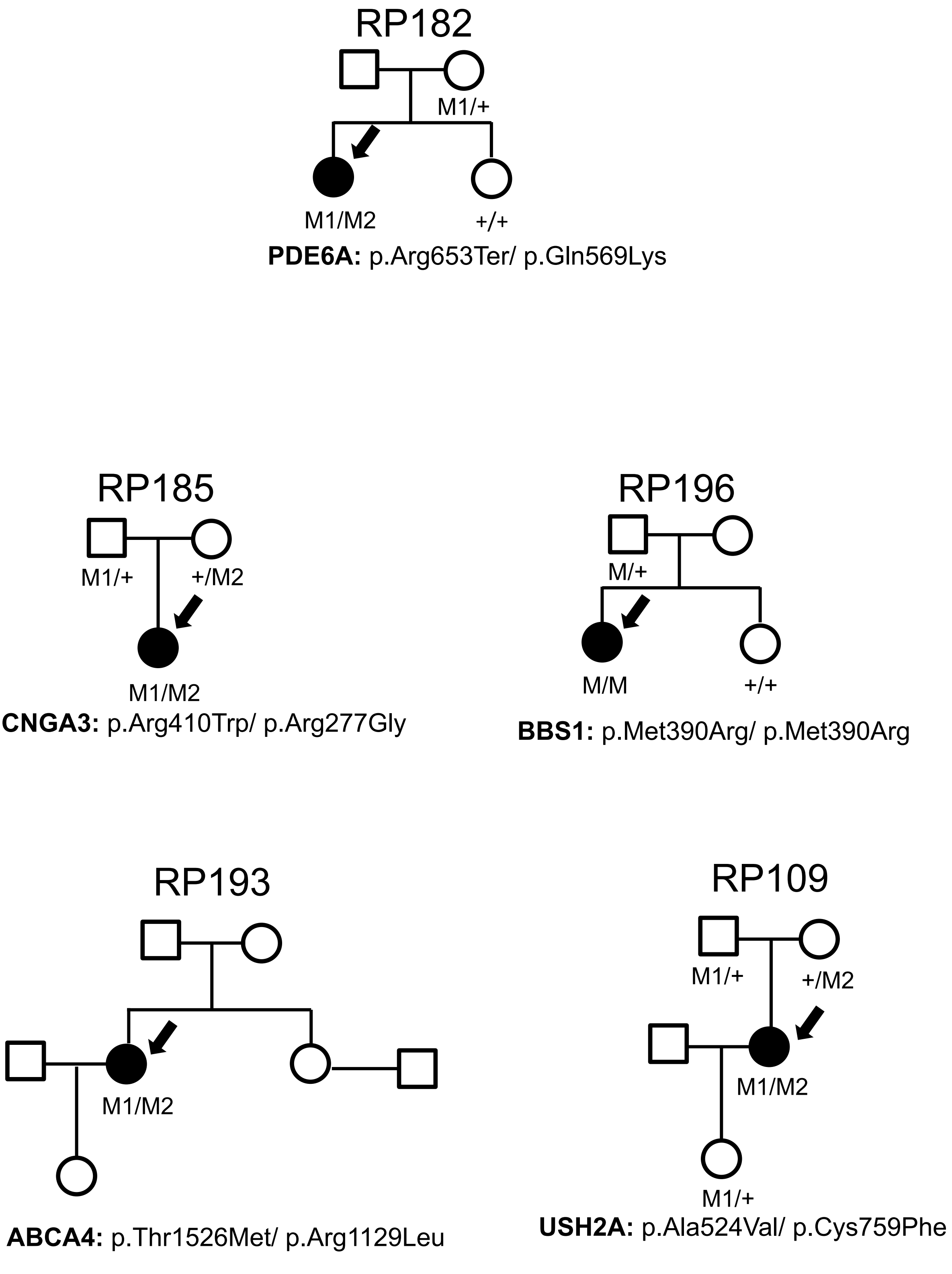
RP182
M1/+
M1/M2
+/+
PDE6A: p.Arg653Ter/ p.Gln569Lys
RP185
M1/+
+/M2
M1/M2
CNGA3: p.Arg410Trp/ p.Arg277Gly
RP196
M/+
M/M
+/+
BBS1: p.Met390Arg/ p.Met390Arg
RP193
M1/M2
ABCA4: p.Thr1526Met/ p.Arg1129Leu
RP109
M1/+
+/M2
M1/M2
M1/+
USH2A: p.Ala524Val/ p.Cys759Phe

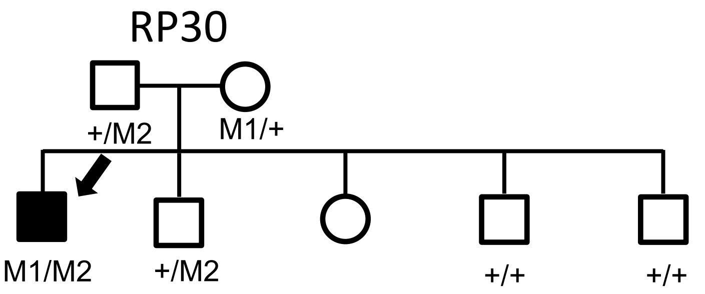

RP1: p.Ser542Ter/ p.Leu76Pro

VUS

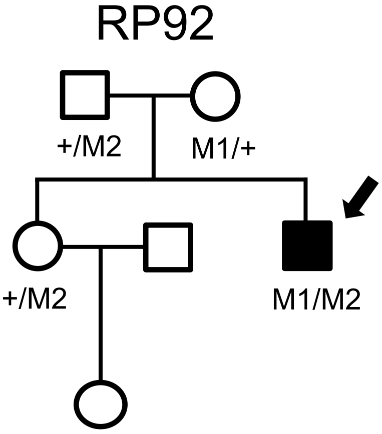

PCDH15/CDH23: p.Arg245Ter / p.Gly2776Ser

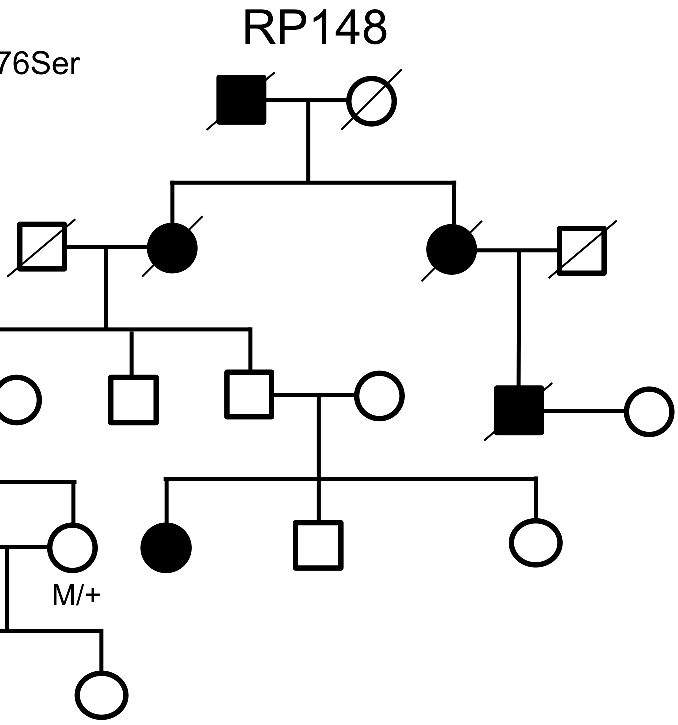

PRPF8: p.Trp2279Gly

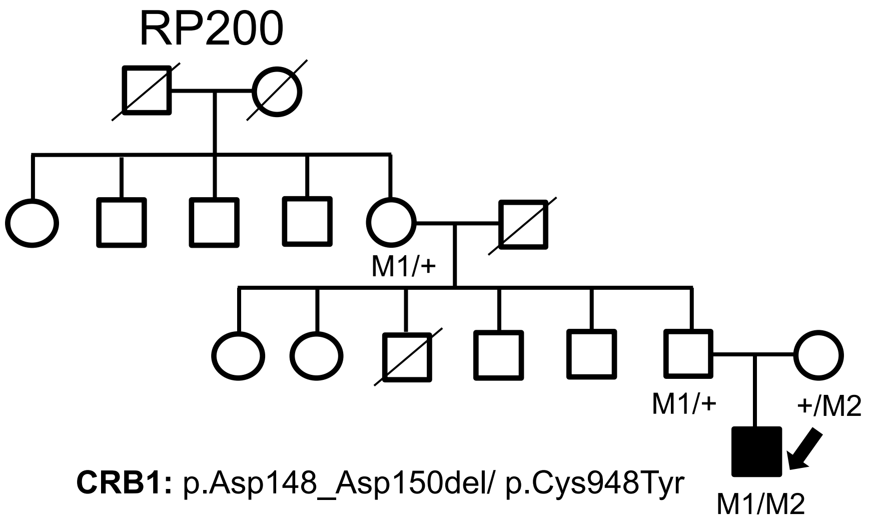
RP200
M1/+
M1/+
+/M2
M1/M2
CRB1: p.Asp148_Asp150del/ p.Cys948Tyr

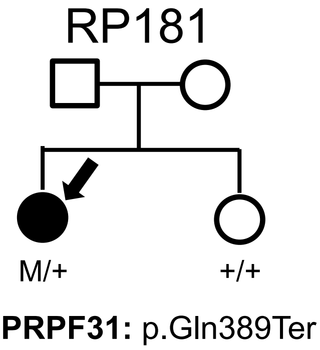
RP181
M/+
+/+
PRPF31: p.Gln389Ter

Supplementary Figure 3

Deletion of 4bp in the ORF15 region of RPGR gene as seen by Integrative Genomics Viewer. Despite the highly repetitive purine-rich sequences present in this region, we were able to detect this novel variant using our pooled sequencing strategy. The total number of reads of each nucleotide, as well as the number of deletions or insertions (bottom) is annotated in each of the 4 central tables. Larger table on the right shows the difference in the number of reads of the contiguous nucleotide, with no apparent deletions detected.

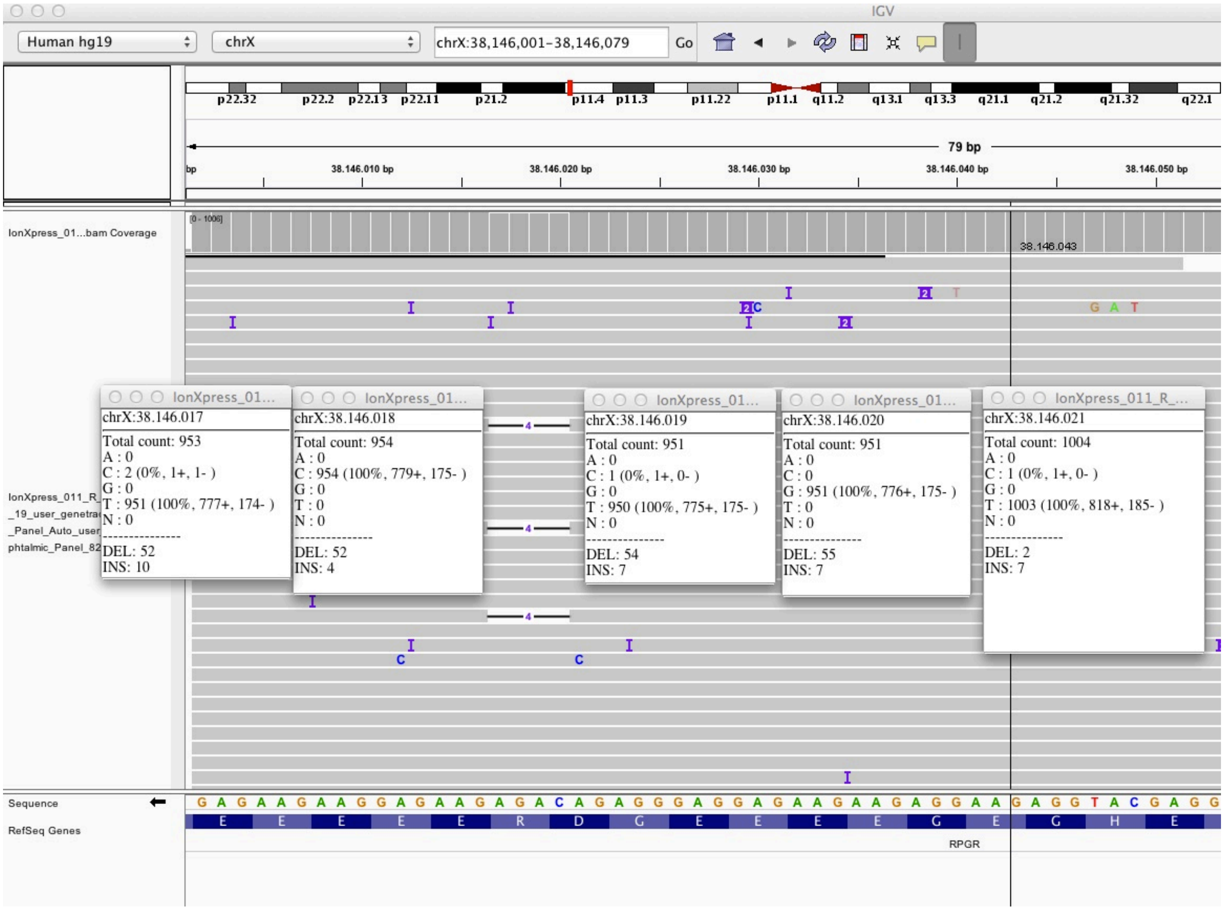

Supplementary Table S1

ABCA4, ABCC6, ABHD12, ACBD5, ACO2, ADAM9, ADAMTS10, ADAMTS18, ADAMTSL4, AGK, AHI1, AIPL1, AKR1E2, ALDH1A3, ALMS1, APOA1, ARL6, ATXN7, B3GALTL, BBS1, BBS10, BBS12, BBS2, BBS4, BBS5, BBS7, BBS9, BCOR, BEST1, BFSP1, BFSP2, BMP4, C10orf2, C12orf57, C1QTNF5, C21orf2, C2orf71, C8orf37, CA4, CABP4, CACNA1F, CACNA2D4, CAPN5, CC2D2A, CDH23, CDH3, CDHR1, CEP164, CEP290, CERKL, CHD7, CHM, CHMP4B, CHN1, CHRDL1, CHST6, CIB2, CLN3, CLRN1, CNBP, CNGA1, CNGA3, CNGB1, CNGB3, CNNM4, COL11A1, COL2A1, COL8A2, COL9A1, CRB1, CRX, CRYAA, CRYAB, CRYBA1, CRYBA4, CRYBB1, CRYBB2, CRYBB3, CRYGB, CRYGC, CRYGD, CRYGS, CSAD, CTDP1, CYP1B1, CYP27A1, CYP4V2, CYP51A1, DCN, DFNB31, DHDDS, DMD, DMPK, DTHD1, EFEMP1, ELOVL4, EMC1, EPHA2, EYS, FAM161A, FLVCR1, FOXC1, FOXE3, FRMD7, FSCN2, FYCO1, FZD4, GALK1, GALT, GCNT2, GDF3, GDF6, GJA3, GJA8, GNAT1, GNAT2, GNPTG, GPR125, GPR143, GPR179, GPR98, GRK1, GRM6KRT3, GUCA1A, GUCA1B, GUCY2D, HARS, HCCS, HDAC8, HMCN1, HMX1, HOXA1, HSF4, IDH3B, IFT140, IGBP1, IGFBP7, IMPDH1, IMPG2, INPP5E, INVS, IQCB1, IQSEC2, JAG1, KCNJ13, KCNV2, KERA, KIAA1549, KIF11, KIF21A, KLHL7, KRT12, KRT3, LCA5, LEPREL1, LIM2, LRAT, LRIT3, LRP5, LTBP2, LZTFL1, MAF, MAK, MERTK, MFN2, MFRP, MFSD6L, MIP, MIR184, MITF, MKKS, MKS1, MTTP, MYH9, MYO7A, MYOC, NBAS, NDP, NHS, NMNAT1, NPHP1, NPHP3, NPHP4, NR2E3, NRL, NTF4, NYX, OAT, OCRL, OFD1, OPA1, OPA3, OPN1LW, OPN1MW, OPN1SW, OPTN, OTX2, PANK2, PAX2, PAX6, PCDH15, PDE6A, PDE6B, PDE6C, PDE6G, PDE6H, PDZD7, PEX1, PEX2, PEX7, PGK1, PHGDH, PHOX2A, PHYH, PIKFYVE, PITPNM3, PITX2, PITX3, PLA2G5, PLOD3, POLG, POLG2, POMT1, PRCD, PRDM5, PROM1, PRPF3, PRPF31, PRPF6, PRPF8, PRPH2, PRSS56, RAB18, RAB3GAP1, RAB3GAP2, RAX, RAX2, RB1, RBP3, RBP4, RD3, RDH12, RDH5, RGR, RGS9, RGS9BP, RHO, RIMS1, RLBP1, RNLS, ROBO3, ROM1, RP1, RP1L1, RP2, RP9, RPE65, RPGR, RPGRIP1, RPGRIP1L, RRM2B, RS1, RYR1, SAG, SDCCAG8, SEMA4A, SETX, SIL1, SIX6, SLC16A12, SLC24A1, SLC25A4, SLC4A11, SMOC1, SNRNP200, SOX2, SPATA7, STRA6, TACSTD2, TDRD7, TEAD1, TENM3, TGFBI, TIMM8A, TIMP3, TMEM126A, TMEM237, TOPORS, TREX1, TRIM32, TRPM1, TSPAN12 , TTC8, TTPA, TUBB3, TULP1, UBIAD1, UNC119, USH1C, USH1G, USH2A, VAX1, VCAN, VIM, VSX1, VSX2, WDPCP, WDR19, WDR36, WFS1, WRN, ZEB1, ZNF423, ZNF469, ZNF513, ZNF644.

Supplementary Table S1. List of genes analysed.

Supplementary Table S2

rs	Relative number of reads		Total relative number of reads	Expected number of alleles	Confirmed alleles according to zygosity		Confirmed alleles
	**WT**	**Mut**			**Het**	**Hom**	
rs17821448	2109	1123	3232	11.1	7	2	11
rs1801555	3070	706	3776	6	3	2	7
rs11373	5041	1958	6999	9	7	0	7
rs61749605	966	368	1334	8.8	2	2	6
rs1801574	2489	401	2890	4	4	0	4
rs4916685	1016686	686	1702	13	7	3	13
rs6666652	2281	178	2459	2.5	2	1	4
rs624851	1207	585	1792	10.5	9	0	9
rs17403955	928	103	1031	3	2	0	2

Supplementary Table S2. Relative level of coverage in variants with high MAF. We selected a set of 9 SNPs with relative high MAF (ranging from 0.1 to 0.37) from the VCF, in order to assess the relative distribution of sequencing reads across samples. In the table are represented the frequency of both WT and mutated alleles (Relative number of reads), and the combination of both (Total relative number of reads). Since our methodology was based on sequencing DNA pools from 16 samples, we expected a relative level of coverage of 1/32 in samples from heterozygous patients and 2/32 in samples from one homozygous or from two heterozygous patients (Expected number of alleles). However we found some variability between expected and confirmed number of alleles, as identified by Sanger sequencing (Confirmed alleles). Heterozygous and homozygous alleles are represented (confirmed alleles according to zygosity). See discussion section for possible explanations for this variability observed. Abbreviations: Het: heterozygote; Hom: homozygote; MUT: mutated allele; rs: reference SNP ID number; WT: wild-type allele.

Supplementary Table S3

A

GENE	ZYGOSITY	VARIANT	cDNA	PROTEIN CHANGE
BBS1	hom	chr11:66293652	c.1169T>G	p.Met390Arg
ABCA4	het	chr1:94473807	c.5882G>A	p.Gly1961Glu
RHO	het	chr3:129252450	c.937-1G>T	c.937-1G>T
USH2A	het	chr1:216052143	c.8521T>A	p.Trp2841Arg
RP1	het	chr8:55537560	c.1118C>T	p.Thr373Ile
CERKL	hom	chr2:182423344	c.847C>T	p.Arg283Ter
USH2A	het	chr1:216420460	c.2276G>T	p.Cys759Phe
CERKL	het	chr2:182423344	c.847C>T	p.Arg283Ter
PDE6A	hom	chr5:149263074	c.2053G>A	p.Val685Met
ROM1	het	chr11:62382123	c.868del	p.Gln290LysfsTer26
RHO	het	chr3:129247835	c.259C>G	p.Val87Leu
USH2A	hom	chr1:215847862	c.13388G>A	p.Thr4464Ter
PRPF31	het	chr19:54626832	c.770-1C>T	c.770-1C>T
PRPF8	het	chr17:1554160	c.6945del	p.Asn2316ThrfsTer43
RP2	hem	chrX:46736931	c.1073-9T>A	c.1073-9T>A

B

GENE	ZYGOSITY	VARIANT	cDNA	PROTEIN CHANGE
USH2A	het	chr1:215932085	c.11241C>A	p.Tyr3747Ter
PRPF31	het	chr19:54621969	c.194T>A	p.Met65Lys
PDE6A	het	chr5:149301194	c.933+4C>T	c.933+4C>T
GUCA1A	het	chr6:42141469	c.118C>T	p.Arg40Cys
PRPF3	het	chr1:150325252	c.2071-57ins	c.2071-57ins
USH2A	het	chr1:216420460	c.2279G>T	p.Cys759Phe
ABCA4	het	chr1:94526230	c.2023G>A	p.Val675Ile
ABCA4	het	chr1:94544977	c.1140T>A	p.Asn380Lys
BEST1	het	chr11:61722590	c.164C>T	p.Thr55Met
BBS10	het	chr12:76739848	c.1917C>G	p.Gly639Gly
RLBP1	hom	chr15:89754954	c.684+20C>T	c.684+20C>T
MERTK	hom	chr2:112740597	c.1296+27del	c.1296+27del
SNRNP200	het	chr2:96959129	c.1957C>A	p.Thr654Asn
RHO	het	chr3:129247887	c.311T>A	p.Val104Asp
RHO	het	chr3:129252535	c.1021G>A	p.Glu341Lys
CNGA1	het	chr4:47938971	c.1747C>T	p.Arg583Ter
CNGA1	het	Chr4:47939328	c.1519C>T	p.Ala459Val
CNGA1	het	chr4:47972953	c.165T>C	p.Ser55Ser
CNGA1	het	chr4:47973110	c.8C>T	p.Ser3Phe
PDE6A	het	chr5:149323876	c.367G>T	p.Asp123Tyr
RPGR	het	chrX:38158349	c.1105C>T	p.Arg369Cys

Supplementary Table S3. **Variants used as positive control in two sets of experiments. A.** pooled DNA with 4, 8 and 16 samples. **B.** 7 pools with 16 samples each. Distribution of control variants among samples is depicted in Supplementary Figure 1.
